# Testing Strategies for Metabolite-Mediated Neurotoxicity

**DOI:** 10.3390/ijms26178338

**Published:** 2025-08-28

**Authors:** Julian Suess, Moritz Reinmoeller, Viktoria Magel, Baiba Gukalova, Edgars Liepinsh, Iain Gardner, Nadine Dreser, Anna-Katharina Holzer, Marcel Leist

**Affiliations:** 1 In Vitro Toxicology and Biomedicine, Dept Inaugurated by the Doerenkamp-Zbinden Foundation, University of Konstanz, 78457 Konstanz, Germany; 2 Latvian Institute of Organic Synthesis, LV-1006 Riga, Latvia; 3 Certara Predictive Technologies, Level 2-Acero, 1 Concourse Way, Sheffield S1 2BJ, UK; 4 Center for Alternatives to Animal Testing in Europe (CAAT-Europe), University of Konstanz, 78457 Konstanz, Germany

**Keywords:** toxicity screening, xenobiotic metabolism, developmental neurotoxicity, neurotoxicity, microsomes

## Abstract

Compounds, which rely on metabolism to exhibit toxicity, pose a challenge for next-generation risk assessment (NGRA). Since many of the currently available non-animal new approach methods (NAMs) lack metabolic activity, their use may lead to an underestimation of the true hazard to humans (false negative predictions). We explored here strategies to deal with metabolite-mediated toxicity in assays for developmental neurotoxicity. First, we present an overview of substances that may serve as potential positive controls for metabolite-related neurotoxicity. Then, we demonstrate, using the MitoMet (UKN4b) assay, which assesses the adverse effects of chemicals on neurites of human neurons, that some metabolites have a higher toxic potency than their parent compound. Next, we designed a strategy to integrate elements of xenobiotic metabolism into assays used for (developmental) neurotoxicity testing. In the first step of this approach, hepatic post-mitochondrial fractions (S9) were used to generate metabolite mixtures (“metabolisation module”). In the second step, these were applied to a NAM (exemplified by the UKN4b assay) to identify metabolite-mediated toxicity. We demonstrate the applicability and transferability of these approaches to other assays, by an exemplary study on the basis of the cMINC (UKN2) assay, another NAM of the developmental neurotoxicity in vitro battery. Based on the experience gained from these experiments, we discuss key issues to be addressed if this approach is to be used more broadly for NAM in the NGRA context.

## 1. Introduction

The toxicity of xenobiotics may be triggered by the compound proper (parent) or any of its metabolites. Most cell systems used as the basis of new approach methodologies (NAMs) have a relatively low xenobiotic metabolising capacity, compared to the human body, and in particular, its liver. Therefore, the testing of parent compounds in NAMs, e.g., those used to assess neurotoxicity or developmental neurotoxicity (DNT), does not necessarily reflect the pattern, and toxicity, of potential mixtures of metabolite plus parent found in human brain tissue.

Next-generation risk assessment (NGRA) aims to predict chemical toxicity in humans by using NAMs. Substantial progress has been made to assemble NGRA frameworks for regulatory decision making in several large collaborative research projects and by several consumer product providers [1,2,3]. A potential shortcoming is the low level of xenobiotic metabolism of most in vitro test systems compared to the in vivo situation. This means that NAM-based risk assessment may underestimate the true toxicity of a chemical to humans (false negative predictions) if metabolites contribute to the toxicity. For several compounds, it is indeed known that metabolism in hepatic and peripheral tissues can form metabolites which differ to a large extent in their toxicodynamic and toxicokinetic properties from the respective parent [4,5,6]. A potential remedy may be strategies that address metabolite-mediated toxicity by incorporating metabolite-generating systems into NAMs. Several modified NAMs, mimicking, e.g., liver, lung, or intestine have been described to capture toxicities linked to reactive metabolites, such as genotoxicity or hapten formation [7,8]. However, multi-organ interactions such as the metabolism of a parent in the liver and the toxic activity of a metabolite in the (developing) nervous system remain a challenge. A particularly well-known examples of metabolite-mediated neurotoxicity are organothiophosphate parents. The parent is metabolised by the liver, and the resultant metabolite blocks signal transduction amongst neurons and at the neuromuscular junction. In such cases, both metabolic activation and the specific biology of the target organ must be represented by the test system to predict the potential hazard [9,10,11].

In the NAM used for neurotoxicity and DNT testing, information about the generation of metabolites from the parent is very limited. Instead, the various NAMs of the DNT in vitro battery (DNT-IVB) have been optimised to reflect key neurodevelopmental processes (KNDPs; [3,12,13,14]). KNDP assays model complex biological processes involving multiple signalling pathways and cellular functions. The assays assess, e.g., neural cell proliferation, differentiation, and neurite outgrowth [12,15,16]. The original DNT-IVB includes the UKN4 (NeuriTox) test. It uses human dopaminergic neurons (LUHMES; [17,18]) and was later optimised to detect mitochondrial toxicants with higher sensitivity (UKN4b assay, MitoMet; [19]). The UKN5 assay (PeriTox) uses a similar test principle but is based on human induced pluripotent stem cell (hiPSC)-derived peripheral neurons [20]. Yet another DNT-IVB assay is the neural crest cell (NCC) migration assay (cMINC; UKN2; [21]). All three NAMs have been widely used in screening campaigns together with regulatory agencies such as the European Food Safety Authority (EFSA) and U.S. Environmental Protection Agency (EPA; [14,18,21,22]). The usefulness and regulatory acceptance of these NAMs would greatly benefit from strategies that integrate metabolic competence.

Several strategies already exist for introducing the effect of (liver) metabolism into in vitro systems. These approaches range from the resource-intensive direct testing of individual metabolites [11,23,24,25,26,27,28] to more technically demanding methods. The latter integrate metabolic competence (metabolic retrofitting) by using microfluidic systems [29,30,31], primary hepatocyte-conditioned media [32,33], recombinant xenobiotic-metabolising enzymes [34], recombinant expression of metabolic enzymes [7,35], and various hepatic subcellular fractions, such as microsomes and post-mitochondrial supernatant (S9), in combination with the original assay design [36,37]. Hepatic S9 fractions have been recommended by experts as the first choice for NAMs, which lack sufficient metabolic capacity for incorporating metabolism into in vitro test systems [38]. For instance, the Ames test employs S9 fractions for mutagenicity screening [39,40,41]. S9 has also been applied in various nuclear receptor assays [37,42,43,44].

The addition of complex biological components and multiple bioactive proteins (as in S9) may affect the performance of some NAMs, as some can be heavily affected. For instance, cellular differentiation often requires highly defined biological niches that may be disturbed, and some cell types do not easily tolerate some of the S9 constituents. Such issues have, in the past, limited the widespread metabolic retrofitting of DNT assays to capture potential metabolite toxicity. The present study was undertaken to investigate the feasibility of metabolite studies and to provide an initial proof of principle that some metabolite-dependent toxicity is detectable in neurotoxicity/DNT NAMs. Several potential positive controls for metabolite-mediated toxicity were described to pave the road towards future assay development. Moreover, some examples for the applicability and transferability of an S9 pre-activation workflow for retrofitting in vitro developmental neurotoxicity test systems was explored.

## 2. Results and Discussion

### 2.1. Parent/Metabolite Pairs to Explore Metabolite-Mediated Toxicity

As a first approach to explore metabolite-mediated (developmental) neurotoxicity, we assembled a collection of parent/metabolite pairs with reported metabolite-mediated toxicity (see Table 1). A parent/metabolite pair was included if the metabolite was identified in the literature as a (developmental) neurotoxicant or when its supposed mode of action was plausibly linked to (developmental) neurotoxicity. An example of the latter are metabolites which are reported as cytoskeletal or mitochondrial toxicants. Compounds with such a mode of action have frequently been observed as hits during in vitro DNT screening [14,17,19,22,45,46,47,48]. We excluded in this section acetylcholinesterase inhibitors or their thiophospho “prodrugs”, as they have already been extensively characterised and do not require sophisticated cell function assays to be identified. We added to this compound selection information relevant to the planning of cell culture-based testing (e.g., some physchem properties; Table 1; Supplementary File S1). Moreover, we used this parent/metabolite collection for an initial exploration of metabolite toxicity in an exemplary human cell-based neurotoxicity assay (see below). Activities by us and other laboratories may expand this initial collection and associated test data in the future.

### 2.2. Strategy to Compare Potency of Metabolites to That of Their Cognate Parents

We based our test strategy on findings well established in the field: First, many approaches have already been developed to predict, identify and quantify metabolites in humans, animals and e.g., hepatocyte cultures [49,50,51,52,53]. Second, some metabolites are clearly bioactive, and their potency, relative to that of the respective parent, is of toxicological significance. We suggest that information from DNT-IVB assays on the relative hazard potential of parent/metabolite pairs is useful for NGRA.

**Figure 1:**
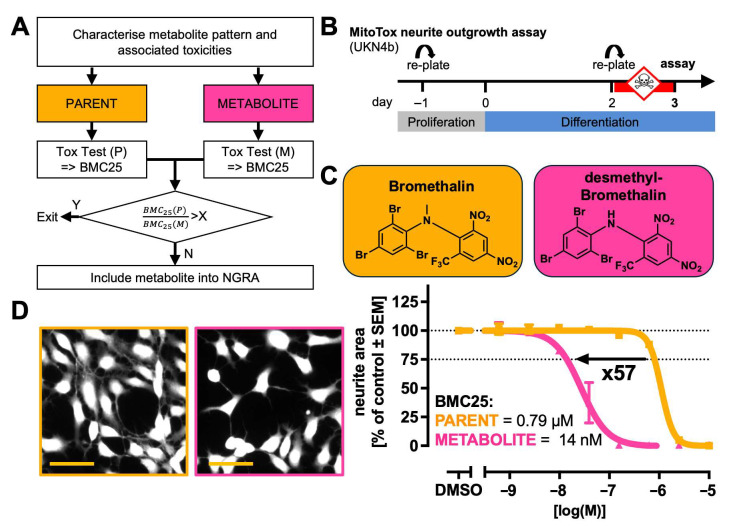
(**A**) A strategy scheme to assess metabolite-mediated hazard in NAMs used for neurotoxicity or developmental neurotoxicity. Parent and metabolite compounds are tested separately in the respective assay. This strategy is applicable when there is prior knowledge of metabolite formation. A decision rule based on the relative toxicities (X) of the parent and metabolite may be applied to decide whether next-generation risk assessment (NGRA) should be applied to the metabolite. The value of X may be defined according to the problem formulation and the NAM used. (**B**) An assay scheme of the neurite outgrowth assay used here for exemplification: Proliferating LUHMES cells were replated one day before differentiation was initiated (day −1). After two days (day 2) in differentiation medium, the cells were replated into assay format (96-well). At this point, neurite growth was re-initiated. The cells were treated with toxicants 1 h after replating. Neurite outgrowth and viability were determined 24 h later, using automated high content imaging. (**C**) Comparison of neurite outgrowth of cultures treated with bromethalin and desmethyl-bromethalin one exemplary parent/metabolite pair (chemical structures shown). The parent compound is displayed in orange (light grey), and the metabolite information is shown in magenta (dark grey). The curves are based on data from three independent experiments and were fitted using a four-parameter Hill function. Data points shown are arithmetic means of data from independent experiments ±SEM. In each independent experiment, the technical replicates were measured and averaged. A benchmark response (BMR) of 25% was used as specified in the detailed assay description, and the respective benchmark concentration (BMC25) was determined. The bold numbers indicate the x-fold change between BMC25 values of the parent and metabolite, with the direction of the arrow reflecting the shift in sensitivity. (**D**) Microscopic images of calcein-stained LUHMES cells. Similar images are used for neurite quantification. Treatment with 40 nM of the parent (left) or metabolite (right). The scale bar in orange corresponds to 50 µm.

Comparing the potency of a metabolite to that of its parent allows for initial classification of its toxicological relevance and forms a basis for a more sophisticated follow-up strategy. For instance, a metabolite exceeding a certain relative potency level could or should be included into NGRA (Figure 1A). The relative potency cut-off (designated X in Figure 1A) depends on the risk assessment problem formulation and on some toxicokinetic properties of the metabolite and parent. For instance, X = 0.1 may be considered when the metabolite is very stable (low clearance) compared to the parent. A cut off with X >> 1 may be relevant, if the metabolite is likely to be quickly cleared or if only a small fraction is reaching the potential target tissue. Besides the relative clearance and barrier crossing of the metabolite vs. parent, differential protein binding may also play a role. This parameter determines the free fraction and is therefore an important information for toxicokinetic modelling. Therefore, we assembled such information (Table 1; Supplementary File S1).

In all cases, obtaining comparative potency data forms the basis for all further activities and decisions. We exemplified the test procedure with bromethalin (parent) and its reported metabolite desmethyl-bromethalin, using the MitoMet neurite outgrowth assay (Figure 1B).

**Table 1:** An overview of the parent/metabolite pairs used to explore metabolite-mediated toxicity. The table summarises parent compounds and their metabolites of concern, including CAS numbers and molecular weights. Free fractions are predicted for human plasma and cell culture medium (fu). Additional comments provide context or specific considerations for the use of the respective compound. The literature references identify data sources.

Parent/CAS MW in g/mol	Comment	Free Fraction [%]		Metabolite of Concern CAS/MW in g/mol	Free fraction [%]		Literature Ref.
		Plasma	Medium		Plasma	Medium	
**Artesunate** CAS: 88495-63-0 MW: 384.4	Prodrug (human), which induces oxidative stress	3.1	98.2	**Dihydroartemisinin** CAS: 71939-50-9 MW: 284.4	14.9	99.7	[54,55]
**Benomyl** CAS: 17804-35-2 MW: 290.3	Pesticide; cytoskeletal toxicant	14.1	99.6	**Carbendazim** CAS: 10605-21-7 MW: 191.2	75.0	100.0	[3,56,57]
**Benzophenone** CAS: 119-61-9 MW: 182.2	Preservative, metabolite is mitochondrial toxicant	93.0	99.4	**4-Hydroxybenzophenone** CAS: 1137-42-4 MW: 198.2	6.8	99.2	[58,59,60]
**Bromethalin** CAS: 63333-35-7 MW: 577.9	Pro-pesticide, metabolite is mitochondrial toxicant	0.3	83.2	**Desmethyl-bromethalin** CAS: 57729-86-9 MW: 563.9	0.1	62.6	[61,62]
**Chlorfenapyr** CAS: 122453-73-0 MW: 407.6	Pro-pesticide, metabolite is mitochondrial toxicant	1.6	96.4	**Tralopyril** CAS: 122454-29-9 MW: 384.4	3.5	98.4	[61,63]
**Febantel** CAS: 58306-30-2 MW: 446.5	Prodrug (veterinary); which is used as cytoskeletal toxicant.	6.0	99.1	**Fenbendazole** CAS: 43210-67-9 MW: 299.3	75.0	100.0	[64,65]
**Haloperidol** CAS: 52-86-8 MW: 375.9	Drug (human), metabolite is reported neurotoxicant	24.5	99.8	**HPP+** CAS: 125785-69-5 MW: 354.8	52.7	99.9	[66,67]
**MDMA** CAS: 64057-70-1 MW: 193.3	Recreational drug, metabolite reported as neurotoxicant	61.7	100.0	**α-Methyldopamine** CAS: 555-64-6 MW: 167.2	88.8	100.0	[68]
**MPTP** CAS: 28289-54-5 MW: 173.3	Recreational drug impurity, metabolite reported as neurotoxicant	28.6	99.9	**MPP+** CAS: 48134-75-4 MW: 170.3	91.1	100.0	[25,69]

Abbreviations: HPP+, haloperidol pyridinium ion; MPP+, 1-methyl-4-phenylpyridinium; MPTP, 1-methyl-4-phenyl-tetrahydropyridine; MDMA, methylenedioxymethamphetamine.

Concentration–response data for effects on the neurite area were obtained for both the parent and the metabolite (Figure 1C). Using the assay’s standard benchmark response (BMR) of 25%, we derived a benchmark concentration (BMC25) for the parent of 790 nM and for the metabolite of 14 nM. Thus, the potency ratio was 57 (parent divided by metabolite). A clear reduction in the neurite area was observed in LUHMES cells treated with desmethyl-bromethalin compared to bromethalin at equimolar concentrations (Figure 1D). The data obtained suggest that the demethylation of bromethalin leads to an increase in potency (with respect to neurite toxicity). This finding agrees with previous reports [61,62] and highlights that some metabolites have a high hazard potential and should not be neglected in NGRA.

### 2.3. Comparison of Parent and Metabolite Potencies in Exemplary Assays

**Figure 2:**
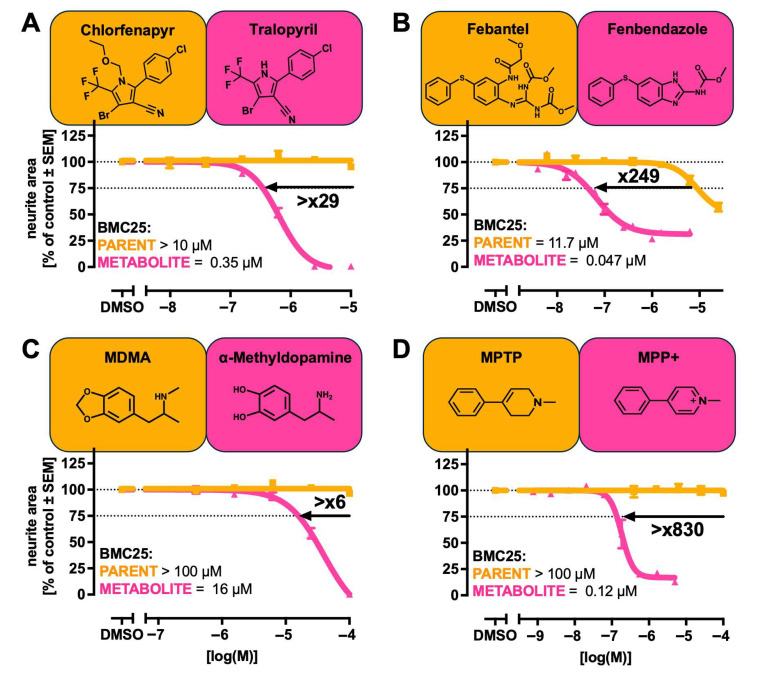
Neurotoxicity hazard comparison of known parent/metabolite pairs. The concentration-dependent effects of a parent compound and a cognate metabolite on neurite outgrowth were determined as in Figure 1. Parent compounds are displayed in orange and metabolites in magenta. The BMC25 values were determined as in Figure 1. Bold numbers indicate the fold change between BMC25 values of the parent and metabolite, with the direction of the arrow reflecting the shift in sensitivity. (**A**) Chlofenapyr and its metabolite tralopyril. (**B**) Febantel and its metabolite fenbendazole. (**C**) MDMA and its metabolite α-methyldopamine. (**D**) MPTP and its metabolite MPP+. Abbreviations: MDMA, methylenedioxymethamphetamine; MPP+, 1-methyl-4-phenylpyridinium; MPTP, 1-methyl-4-phenyl-tetrahydro-pyridine.

**Figure 3:**
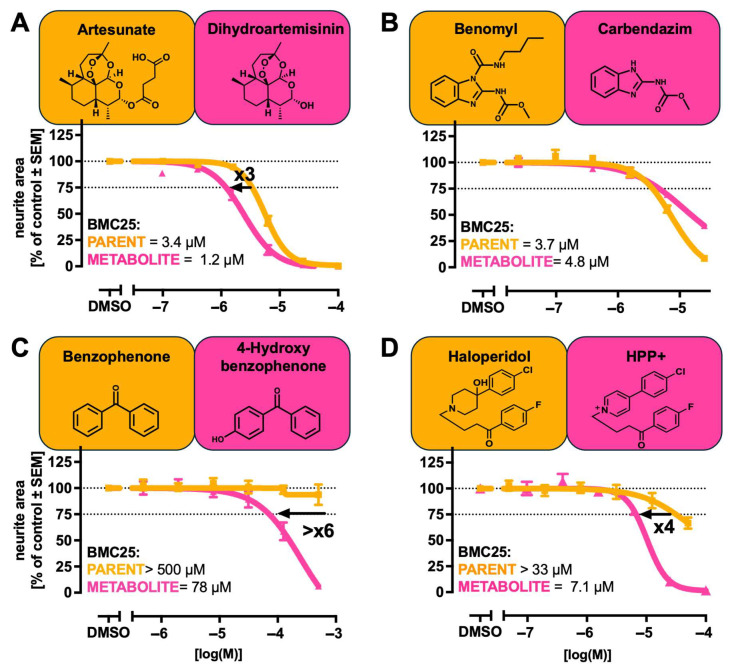
A comparison of the neurotoxicity hazard of additional parent/metabolite pairs. The concentration-dependent effects of a parent compound and a cognate metabolite on neurite outgrowth were determined as in Figure 1. Parent compounds are displayed in orange and metabolites in magenta. The BMC25 values were determined as in Figure 1. Bold numbers indicate the fold change between BMC25 values of the parent and metabolite, with the direction of the arrow reflecting the shift in sensitivity. (**A**) Artesunate and its metabolite dihydroartemisinin. (**B**) Benomyl and its metabolite carbendazim. (**C**) Benzophenone and its metabolite 4-hydroxybenzophenone. (**D**) Haloperidol and its metabolite HPP+. Abbreviations: HPP+, haloperidol pyridinium ion.

We determined the BMC25 for inhibiting neurite outgrowth in LUHMES cells (MitoMet assay) for a further eight parent/metabolite pairs. From these data, we calculated the potency ratios between the parent and metabolite and visualised the shifts in potency (Figure 2 and Figure 3). All metabolites tested exhibited an inhibitory effect on neurite outgrowth. However, the magnitude of the potency shifts between the parent and metabolite varied widely. For example, MPP+ was found to be 830-fold more potent than MPTP, fenbendazole was about 250-fold more potent than febantel, and tralopyril exhibited more than a 29-fold increase in potency compared to chlorfenapyr. For the active metabolites 4-hydroxybenzophenone (>6x more potent than benzophenone), α-methyldopamine (>6 more potent than MDMA), and HPP+ (4x more potent than haloperidol), the effect was less pronounced but still highly relevant. Some metabolites were bioactive, but not significantly more potent than their parents. For example, dihydroartemisinin (3x compared to artesunate) and carbendazim (0.77x compared to benomyl) belong to this group. These findings suggest that comparative parent/metabolite testing may be used to generate data that help to decide on more extensive metabolite assessment routines within NGRA approaches. On this basis, metabolites with comparatively low bioactivity may be excluded from further investigation. Conversely, metabolites with very high bioactivity would be prioritised for further scrutiny.

In the next step, we checked whether the proof-of-concept approach with the MitoMet assay may be transferred to other DNT/neurotoxicity tests. A set of four parent/metabolite pairs was tested in the PeriTox [20] assay, i.e., for interference with human peripheral neurite outgrowth (Supplementary Figure S1A). Interestingly, some potency shifts in parent/metabolite pairs in the PeriTox assay differed from those seen in the MitoMet assay. For example, the bromethalin/desmethyl-bromethalin ratio was 3, the chlorfenapyr/tralopyril ratio was 15, the benomyl/ carbendazim ratio was 0.2, and neither benzophenone nor its hydroxy metabolite showed any significant effect (Supplementary Figure S1B).

As the second follow-up, we used the cMINC neural crest migration assay [22]. Here, metabolite bioactivity was assessed for three exemplary parent/metabolite pairs. The activity ratios were as follows: desmethyl-bromethalin exhibited a 44-fold increase in potency compared to bromethalin; tralopyril was 28 times more potent than chlorfenapyr; and 4-hydroxybenzophenone was over three times more potent than benzophenone (Supplementary Figure S2). The shifts were more similar to the MitoMet assay than to the PeriTox assay. The diversity of metabolite shift patterns suggests that a future strategy of metabolite testing in the DNT-IVB should ideally derive data from more than one assay.

### 2.4. Strategy to Incorporate Metabolic Competence into In Vitro DNT Testing Methods

**Figure 4:**
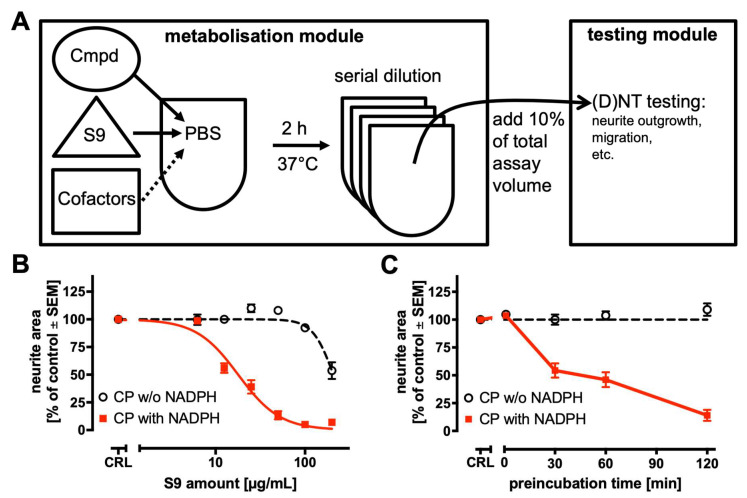
A strategy to incorporate metabolic competence into in vitro DNT testing methods. (**A**) A schematic overview of a sequential metabolism and testing approach. The “metabolisation module” uses hepatic post-mitochondrial fractions (S9) to generate metabolite mixtures in the presence or absence of cofactors (NADPH, glucose-6-phosphate, MgCl_2_). These metabolite mixtures are tested in the “testing module” in a concentration-dependent manner after serial pre-dilutions. Metabolite mixtures are added as a fraction of 10% to the media volume of the “testing module”. The testing module may be a cell-based neurotoxicity (NT) or developmental neurotoxicity (DNT) assay, such as the neurite outgrowth or migration assay. Here, the Mito-Met neurite outgrowth assay, as in Figure 1, was used. (**B**) Optimising S9 concentrations in the metabolisation module (10x concentrated compared to testing module): 1 mM cyclophosphamide (CP) with or without NADPH was added to different amounts of S9 and incubated for 2 h. The curves represent data from three independent experiments and were fitted using a four-parameter Hill function. (**C**) Optimising preincubation time in the metabolisation module: 1 mM cyclophosphamide (CP) was added to 500 µg/mL S9 (with and without NADPH) and incubated for the indicated times. The data represent three independent experiments. The indicated concentrations are “testing module” concentrations.

Having obtained evidence that DNT-IVB assays can identify metabolite bioactivities and give information on their relative potency (compared to cognate parents), we tested approaches to generate metabolites directly within a DNT testing strategy. Several options were explored and evaluated for technical feasibility and practical applicability. The only approach working robustly in our hands was a two-step process of sequential metabolism and testing (Figure 4A). In this strategy, the “metabolisation module” uses hepatic post-mitochondrial fractions (S9) to generate metabolite mixtures in the presence or absence of cofactors (NADPH, glucose-6-phosphate, MgCl_2_) required for the enzymatic reactions. These metabolite mixtures are subsequently transferred into a separate “testing module”. Similar strategies have been reported by others [33,43,70]. During the pilot testing phase, we found that a key challenge in using S9 is the potential cytotoxicity of S9 to highly sensitive test systems used in the DNT-IVB. However, low, non-cytotoxic concentrations of S9 often result in insufficient metabolic activity. Our modular pre-activation approach allows the use of high S9 concentrations in the “metabolisation module”. During toxicity testing, this fraction is diluted to mitigate the potential cytotoxicity of S9. For experiments aimed at the setup and optimisation of the “metabolisation module”, we used cyclophosphamide as the model compound. This prototypical prodrug is well-known to become cytotoxic upon P450-mediated bioactivation [71]. The MitoMet neurite outgrowth assay was used as the testing module (see Figure 1B). In the first experiments, a fixed concentration of cyclophosphamide (100 µM) was incubated with varying S9 concentrations for 2 h (Figure 4B). High S9 concentrations (>100 µg/mL) inhibited neurite outgrowth independent of NADPH, indicating the intrinsic toxicity of S9. Low S9 concentrations (<10 µg/mL) did not lead to neurite toxicity of cyclophosphamide, suggesting insufficient metabolic capacity. At intermediate S9 concentrations, a clear difference between conditions with and without NADPH was detected. We decided to use the maximal non-cytotoxic concentration for further experiments (50 µg/mL). The experiments indicated that only a narrow window of S9 concentrations can be used. This may have to be adapted to assays used for the testing module.

In the next step, we tested the role and optimal conditions of the metabolisation step. Cyclophosphamide (100 µM) was incubated with 50 µg/mL S9 for varying pre-activation times (Figure 4C). No inhibition of neurite outgrowth was observed without preincubation. The magnitude of neurite outgrowth inhibition progressively increased with longer incubation times in the presence of NADPH. After 2 h preincubation, neurite outgrowth was reduced by approximately 90%. These results demonstrate that (i) preincubation is necessary in this setup, (ii) the metabolic activation reaction does not take place (to a significant degree) after the transfer and dilution of the pre-activated fraction, and (iii) flexible handling of preincubation times is technically feasible and shortened times could be used for investigating metabolites that would be further converted to secondary metabolites with long preincubation times. To summarise, the data obtained with cyclophosphamide suggest that a pre-activation workflow can be used to incorporate metabolic competence into the MitoMet neurite outgrowth assay.

Previous studies using S9 fractions found that metabolic conversion of several substrates occurred predominantly within the first two hours of incubation [43,70]. Based on these findings, combined with data from our own pilot experiments, we selected a 2 h preincubation period for further experiments.

### 2.5. Effect of NADPH-Dependent Metabolism on Neurotoxicity of Selected Compounds

**Figure 5:**
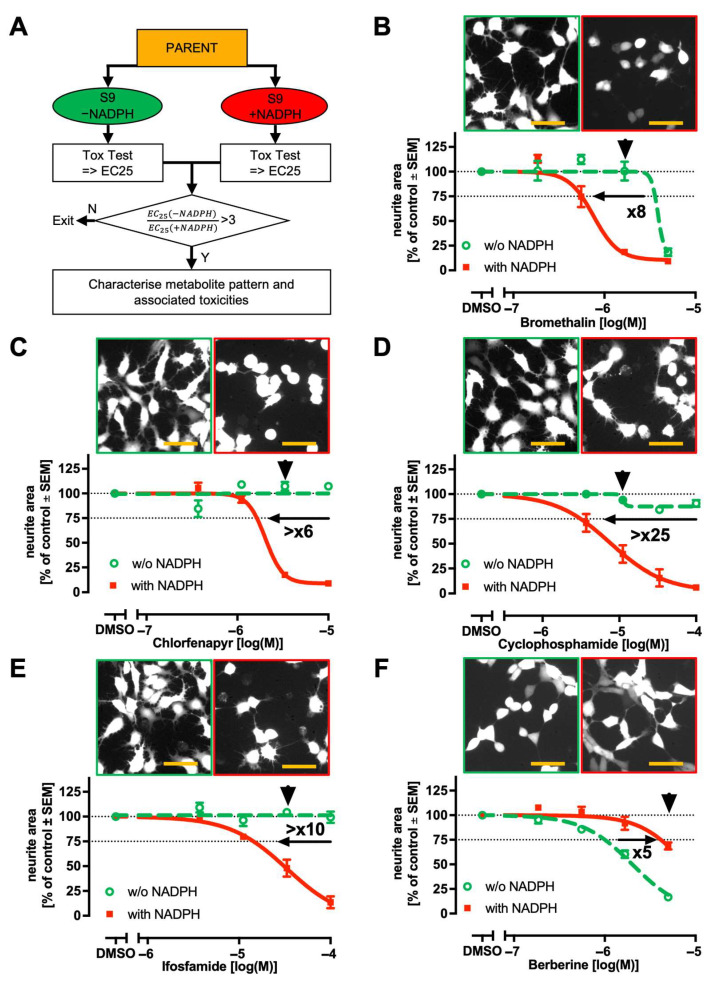
The effect of NADPH-dependent hepatic metabolism on the DNT of selected compounds. (**A**) A strategy scheme to assess the effect of metabolic enzymes on the outcome of cell-based DNT assays. Metabolite mixtures are generated using the NADPH-(in)dependent metabolic activity of S9. NADPH-independent metabolism is shown in green (light grey). NADPH-dependent metabolism is shown in red (dark grey). (**B**–**E**) Examples for NADPH-dependent toxification. (**F**) Example for detoxification. (**B**–**F**) The concentration-dependent effect of metabolite mixtures on neurite outgrowth was measured as in Figure 1, with the following modifications: red-fluorescent-protein (RFP)-expressing LUHMES cells were used. As the assay endpoint, the RFP fluorescence of neurites was used instead of calcein staining. The curves represent data from three independent experiments and were fitted using a four-parameter Hill function. From this, an EC25 was calculated. Bold numbers indicate the fold changes between EC25 values of the NADPH-(in)dependent metabolite mixtures, with the direction of the arrow reflecting the shift in sensitivity. Note that the x-axes indicate nominal concentrations of parent compounds. The images show representative microscopic fields with RFP fluorescence displays. Similar images were used for neurite quantification. The right image (red frame) of the pairs was obtained with NADPH. The black arrowheads above the curves indicate the experimental conditions used to record images. The scale bar in orange corresponds to 50 µm.

To further evaluate our modular pre-activation strategy for identifying metabolite-mediated hazard, we tested a broader panel of compounds. For each compound (parent), a single concentration was used in the metabolisation module. The metabolite mixtures were generated in the absence or presence of NADPH. These metabolite mixtures were serially diluted and assessed using the MitoMet neurite outgrowth assay. As a measure of potency, EC25 values were determined, and potency ratios were calculated to compare the effects of NADPH-dependent versus NADPH-independent metabolism. Exemplary shifts in potency were visualised (Figure 5A).

For four compounds, cells were much more sensitive to neurite damage if treated with metabolite mixtures that were generated in the presence of NADPH (Figure 5B–E). Metabolisation increased the toxicity of the two related prodrugs, cyclophosphamide (>25x) and ifosfamide (>10x), as well as of the pro-pesticides bromethalin (8x) and chlorfenapyr (>6x). The findings are in line with the general assumption that the CYP enzymes of S9 fractions are only enzymatically active when NADPH is present as the electron donor. This suggests that our test strategy allows for the detection of metabolites, which are generated by CYP metabolism.

For a final set of metabolisation experiments, we selected a compound known to be toxic for neurites but also firmly established to be inactivated by gastrointestinal and liver metabolism. Berberine fulfils these conditions [72,73]. It acts as a mitochondrial toxicant by inhibiting complex I [19,74] and is frequently used as a positive control for NAMs, e.g., in cell painting assays [75]. In agreement with our test hypothesis, we found that the toxicity of berberine was reduced upon metabolisation in the presence of NADPH (Figure 5F).

Our data showed how metabolic competence may be inserted into DNT assays, using a modular pre-activation strategy. However, at its current state, this approach has some limitations: it is strongly focused on NADPH-dependent metabolic transformations, which means that parent/metabolite relationships arising from NADPH-independent pathways may be missed.

To better understand which conversions are NADPH-dependent, we measured parent compound degradation and key metabolite formation in the presence and absence of NADPH (Supplementary Figure S3). For the tested parent/metabolite pairs (selection based on technical feasibility), MPTP to MPP+, artesunate to dihydroartemisinin, benzophenone to 4-hydroxybenzophenone, chlorfenapyr to tralopyril, and haloperidol to HPP+, a time-dependent increase in metabolite formation was observed. As this experiment was performed with human liver microsomes, it provides evidence that the selected metabolite may indeed be formed in humans. However, the experiment showed that the extent of metabolite formation varied considerably. Artesunate was completely converted to dihydroartemisinin. For MPP+ and tralopyril, the conversion rate was approximately 10 percent. In contrast, only trace amounts of 4-hydroxybenzophenone and HPP+ were detected. Regarding cofactor dependence, tralopyril formation was clearly dependent on NADPH, indicating CYP involvement. In contrast, the formation of dihydroartemisinin and MPP+ occurred largely independently of NADPH, suggesting alternative enzymatic pathways such as esterases or monoamine oxidases.

The general idea of comparing the toxicity of compounds after incubation at conditions that allow for metabolic conversion is not new. We suggest here the particular use of this strategy to flag compounds that may trigger/enhance DNT after metabolic conversion. If a compound is flagged this way, we suggest that its metabolite pattern and associated toxicities should be characterised in-depth (Figure 5A). For the specific example of the MitoMet assay, we suggest flagging compounds for which NADPH increases toxicity 3-fold in the presence of S9. It is important to note that the toxicity of metabolite mixtures depends on the effects of the residual parent, in addition to one or several metabolites.

### 2.6. Transfer of Metabolisation Module to Neural Crest Cell Migration Assay (cMINC)

**Figure 6:**
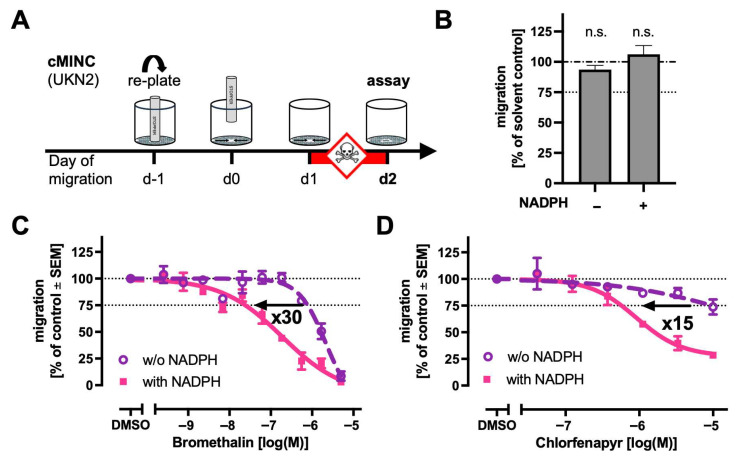
The transfer of the metabolisation module to the neural crest cell migration assay (cMINC). (**A**) A schematic of the cMINC (circular migration of neural crest cells) assay. The cells were seeded around a central stopper to create a cell-free circular area. Migration was initiated 24 h after seeding by removing the stopper. This is defined as the day of migration (DoM) 0. On DoM 1, the cells were exposed to toxicants. After 24 h of exposure, both cell migration and viability were quantified, using automated high-content imaging. (**B**) Compatibility of the metabolisation module (preincubation with 500 µg/mL S9 at 37 °C for 2 h) with the cMINC assay: The setup was as described in Figure 4 and as exemplified in Figure 5A. No compound was added (solvent control). The S9 mixture (at 500 µg/mL ± NADPH) was added to the normal cell culture medium of the cMINC assay (so that the addition was 10% of the total volume). Data are from three independent experiments. (**C**,**D**) The concentration-dependent effects on migration were evaluated for metabolite mixtures generated from bromethalin (**C**) or chlorfenapyr (**D**). The curves represent data from three independent experiments and were fitted using a four-parameter Hill function. The arrows indicate the direction of sensitivity shift between NADPH-dependent and -independent metabolite mixtures, with accompanying fold change values. The concentrations are given as nominal concentrations of the parent compound. Abbreviations: n.s., not significant.

One advantage of a modular metabolisation approach is its potential transferability to different in vitro assays. For instance, a single metabolite mixture, once generated, could be tested across various DNT assays. We selected here the cMINC assay (UKN2; Figure 6A) to test the transfer of the metabolisation module. First, we checked the general compatibility of the two modules. The addition of the metabolisation mixture (without any test compound) did not significantly alter the general assay parameters of the cMINC (Figure 6B). Second, we tested metabolite mixtures derived from the pro-pesticides bromethalin and chlorfenapyr. The bromethalin potency increased about 30-fold from an EC25 of 790 nM (−NADPH) to 26 nM (+NADPH). Chlorfenapyr’s potency shifted 15-fold from an EC25 of 9 µM (−NADPH) to 0.6 µM (+NADPH; Figure 6C,D). These shifts are different from those observed in the MitoMet assay. These results suggest (i) that the metabolisation module can in principle be transferred to a different in vitro assay and (ii) that metabolite testing in the DNT-IVB should ideally derive data from more than one assay.

## 3. Materials and Methods

### 3.1. Materials

Unless specified otherwise, cell culture reagents (consumables and media) were from Gibco/Thermo Fisher Scientific (Waltham, MA, USA), and fine chemicals (inhibitors, substrates) were from Sigma-Aldrich (Steinheim, Germany). Physicochemical properties, CAS identifiers, and literature references of the tested set of chemicals are compiled in Supplementary File S1.

### 3.2. MitoMet Neurite Outgrowth Assay

The MitoMet neurite outgrowth assay was performed as previously described [19,76]. In brief, all cell culture dishes and flasks were pre-coated with 50 μg/mL poly-l-ornithine (PLO) and 1 μg/mL fibronectin. Proliferating LUHMES cells were maintained in AdvDMEM/F12 containing L-glutamine (2 mM), N2 supplement (1x), and FGF (40 ng/mL). For pre-differentiation, the cells were replated at a density of 46,000 cells/cm^2^ one day prior to the induction of differentiation (defined as the day of differentiation, Day −1). Differentiation was initiated by exchanging the culture medium for AdvDMEM/F12 supplemented with L-glutamine (2 mM), N2 supplement (1x), dibutyryl-cAMP (1 mM), tetracycline (1 µg/mL), and GDNF (2 ng/mL). After two days of differentiation (Day 2), the cells were replated (100,000 cells/cm^2^) into 96-well plates. The final assay medium consisted of glucose-free AdvDMEM/F12 supplemented with galactose (18 mM), L-glutamine (2 mM), and tetracycline (1 µg/mL). One hour after replating, the cells were treated with a 10× concentrated stock solution of the test compound. Neurite outgrowth and viability were determined 24 h later, using automated high content imaging.

### 3.3. PeriTox Neurite Outgrowth Assay

PeriTox assay was performed as previously described [20]. In brief, cryopreserved hiPSC-derived immature human dorsal root ganglia neuron-like cells were thawed and plated at a density of 100,000 cells/cm^2^ into matrigel-coated 96-well plates. The cells were cultured in 75 µL PeriTox differentiation medium (PDM) consisting of 25% KSR-S and 75% N2-S media supplemented with 1.5 µM CHIR99021, 1.5 µM SU5402, and 5 µM DAPT (KSR-S: knockout DMEM with 15% serum replacement, 1x Glutamax, 1x nonessential amino acids, and 50 mM beta-mercaptoethanol; N2-S: DMEM/F12, with 2 mM Glutamax, 0.1 mg/mL apotransferrin, 1.55 mg/mL glucose, 25 µg/mL insulin, 100 mM putrescine, 30 nM selenium, and 20 nM progesterone). After one hour, 25 µL PDM with 4x concentrated serial dilutions of the test compounds was added to the cells. Neurite outgrowth and viability were determined 24 h later, using automated high-content imaging

### 3.4. Image Acquisition and Quantification in Neurite Assays

The image acquisition and analysis were conducted according to established protocols [17,77]. The cells were stained with Hoechst H-33342 (1 µg/mL) to visualise nuclei and calcein-AM (1 µM) to label viable cell structures. Fluorescent images were acquired using an automated imaging system. An image analysis algorithm detected nuclei to define somatic regions, which were then excluded from the total calcein-positive area. The remaining signal was quantified as the neurite area.

### 3.5. Neural Crest Cell Migration Assay (cMINC)

The MINC assay was performed as previously described [22]. Briefly, 96-well plates were prepared by inserting a central 2 mm diameter stopper (Platypus Technologies, Madison, WI, USA) into pre-coated wells with a composite poly-L-ornithine (PLO; 10 µg/mL), fibronectin (1 µg/mL), and laminin (1 µg/mL) matrix. Cryopreserved human induced pluripotent stem cell (hiPSC)-derived neural crest-like cells were thawed and seeded at a density of 95,000 cells/cm^2^ around the stopper to create a defined, cell-free circular area. The cells were cultured in N2-S medium supplemented with 20 µg/mL of the cytokines EGF and FGF. After 24 h, the stoppers were removed and the medium refreshed to initiate cell migration into the cell-free centre (defined as the day of migration, DoM 0). On DoM 1, the cells were exposed to test compounds by the addition of a 5× concentrated toxicant solution. After a further 24 h (DoM 2), cell migration and viability were assessed. The cells were stained with 0.5 µM calcein-AM and 1 µg/mL Hoechst 33342 (both from Sigma-Aldrich, Steinheim, Germany). High content imaging was performed, and data were analysed using the RingAssay V1 software (http://invitrotox.uni-konstanz.de/, accessed on 3 February 2025).

### 3.6. Data Analysis: Curve Fitting and Deriving BMC and EC Values

All assays were performed at least three times (biological replicates), with each run evaluating three technical replicates, i.e., different wells with similar treatment. The measured endpoints (migration and neurite area) are expressed relative to the solvent control. In the first step, matched technical replicates were averaged. Subsequently, these data were averaged across the different experiments. Curve fitting was performed employing a 4-parameter log-logistic function with least squares fit. The upper asymptote of the fit was forced to 100%; the lower asymptote was variable. For visualisation, GraphPad Prism 9 was used. For the calculation of the benchmark concentration values (BMC), the online tool BMCeasy V1 (http://invitrotox.uni-konstanz.de/BMCeasy/, accessed on 3 March 2025) was employed [78].

### 3.7. Preincubation of Compound with S9

The “metabolisation module” used hepatic post-mitochondrial fractions (S9) from phenobarbital/β-naphthoflavone-induced male Sprague Dawley rat liver at a final concentration of 500 µg/mL unless otherwise stated. Cryopreserved S9 (MolTox) was thawed, diluted in PBS, and centrifuged at 10,000× *g* for 10 min at 4 °C. The supernatant was used further. Test compounds were first fully dissolved in DMSO and prepared as 1000× concentrated stock solutions and then added as a fraction of 1% (*v*/*v*) to the S9 preparation. Incubation was performed with cofactors required for enzymatic activity (0.8 mM NADPH, 3 mM glucose-6-phosphate, and 5 mM MgCl_2_), or in cofactor-free conditions, at 37 °C for 2 h in sealed deep-well plates, unless stated otherwise. After incubation, the metabolite mixtures were serially pre-diluted with PBS; S9 and cofactors were kept at a constant concentration. These metabolite mixtures were added as a fraction of 10% to the media volume of the “testing module”. The testing module may be a cell-based neurotoxicity assay.

### 3.8. Microsomal Stability Assay

Parent compounds were incubated at final concentrations of either (a) 5 µM or (b) 50 µM (for chlorfenapyr) with pooled human liver microsomes (HLM; mixed gender, n = 150; BioIVT; West Sussex, UK) at corresponding protein concentrations of (a) 0.52 mg/mL or (b) 5.2 mg/mL. Incubations were performed in the absence or presence of NADPH at final concentrations of (a) 1 mM or (b) 10 mM, respectively, using a thermostated orbital shaker at 37 °C and 150 rpm. Reactions were terminated at designated time points by the addition of acetonitrile/methanol (3:1, *v*/*v*). Samples were then centrifuged at 10,000× *g* for 10 min, and the resulting supernatants were analysed by LC-MS/MS.

### 3.9. Liquid Chromatography–Tandem Mass Spectrometry Analysis

All analytes were measured using an Acquity UPLC H-Class system coupled to a Xevo TQ-S micro mass spectrometer (Waters). Chromatographic separation was performed on an Acquity UPLC BEH C18 column (2.1 × 50 mm, 1.7 µm) with a mobile phase consisting of 0.1% formic acid in water (A) and acetonitrile (B). Unless otherwise stated, the column was maintained at 30 °C and operated at a flow rate of 0.4 mL/min. The standard gradient was 95% A from 0 to 1.0 min, decreased to 2% A by 1.5 min, held until 4.0 min, returned to 95% A at 4.3 min, and held until 5.0 min. For MPTP/MPP+ analysis, the column temperature was set to 50 °C, with a flow rate of 0.45 mL/min, and a modified gradient: 92% A (0–1.3 min), 2% A (1.8–4.0 min), returned to 92% A at 4.3 min, and held until 6.0 min. The mass spectrometer operated in electrospray ionisation (ESI) mode. Compound-specific MRM transitions, cone voltages, and collision energies for all analytes are listed in Supplementary Table S2.

### 3.10. Prediction of Protein Binding

For each compound, predictions of plasma protein binding and binding to the S9 matrix were made. Binding to human plasma was predicted from the physicochemical properties of the compound according to the method described by Lowell and Sivarajah [79]. The KD for binding to albumin was calculated assuming that all of the binding in the plasma was due to binding to albumin. The calculated KD value was then used to calculate the binding within the in vitro system to Albumax, accounting for the difference in concentration of albumin in the in vitro test system (400 μg/mL) compared to the concentration in plasma (~45 mg/mL). Binding to the microsomal component of S9 was calculated within the Simcyp simulator V24 using the method described by Gardner et al. [80]. It was assumed that the binding to 0.5 mg/mL of the S9 mixture was equivalent to the binding to microsomal incubation at a protein concentration of 0.5 mg/mL. The binding to S9 at 0.05 mg/mL (i.e., following dilution of the mixture into the media of the NAM) was also calculated. Lastly, binding to albumin and diluted S9 components within the NAM media were assumed to be independent events and the final fu in the NAM assay was obtained by multiplication. Using this approach, the predicted fu in the NAM in the presence of Albumax and diluted S9 fraction was <0.8 for only three compounds, bromethalin (fu = 0.21), chlorfenapyr (fu = 0.68), and desmethyl-bromethalin (fu = 0.054).

## 4. Conclusions and Outlook

Post-mitochondrial supernatant (S9) fractions are frequently employed to incorporate metabolic competence into NAMs. The addition of such complex biological components has little effect on the performance of some NAMs. However, functional assays based on complex differentiation protocols and human neuronal cells (as in the DNT-IVB) may be severely disturbed. In this proof-of-concept study, we encountered several problems and developed some practical solutions (Supplementary Table S3), which may support the retrofitting of other cell-based assays with metabolic competence.

To mitigate the potential disturbance of the neuronal test systems by S9 components, we implemented a two-step approach that separates the metabolism and testing phases. In this setup, metabolite mixtures are generated in a dedicated “metabolisation module” and subsequently transferred as a fraction to a separate “testing module”. This decoupling reflects the physiological situation in which metabolites formed in the liver may affect distant tissues such as the (developing) nervous system. It also offers significant technical advantages. These include the flexibility to optimise metabolic conditions (e.g., S9 and cofactor concentrations, buffer composition, and incubation time) without directly impacting cell-based assays. The key challenge encountered was finding the right balance of (high) enzymatic turnover with (low) S9-induced cytotoxicity.

Another factor to be considered is the potential binding of the test compound to S9 protein. This may significantly affect the free concentration [81] of test compounds and the active concentration of metabolites. For our compound selection, this appeared to be a relevant parameter (Supplementary Table S1). To account for this, we consistently compare the parent compound with S9 fractions in the presence and absence of NADPH. However, this restricts the assay applicability to enzymatic conversions which are NADPH-dependent. This focuses the metabolism outcomes mostly on CYP enzymes. CYPs are considered the most relevant class of enzymes for phase I xenobiotic metabolism [52,82,83]. Some estimate that approximately 95% of phase I xenobiotic metabolism is CYP-dependent [83]. Focusing on CYP-mediated activation (also including flavin-containing monooxygenase (FMO)) represents thus a justifiable and practical starting point. Nevertheless, it should be considered that other enzymes may also play a role (e.g., esterases; [83,84,85]). Our pre-activation strategy mirrors in vivo scenarios where stable hepatic metabolites are formed. Short-lived reactive metabolites are unlikely to reach the nervous system at relevant concentrations. Moreover, examples of neurotoxicity caused by phase II metabolites are not known to us.

In this context, one may ask whether other metabolisation modules (e.g., based on human microsomes) may improve test outcomes [86]. There are some advantages of the here used rat S9: first, it was prepared after CYP-induction and thus has a considerably higher activity (required to generate alerts) than standard human S9 preparations; second, it is routinely used for highly standardised in vitro assays (e.g., to assess genotoxicity for drug discovery and regulatory purposes [39,40,41,87,88,89]). Thus, a lot of experience and background data is available. In addition, rat S9 is derived from standardised sources, and has therefore a relatively low variance and high enzymatic activity. Nonetheless, the use of human-derived or biotechnological alternatives [90] may help to address potential limitations in the future.

In the context of the use of metabolisation modules within an NGRA test strategy, it is important to clearly distinguish three situations: (i) the formation of metabolites that do not significantly contribute to toxicity; (ii) the formation of metabolites with a clear toxicity but short half-life and limited distribution in the body; (iii) the formation of stable metabolites that significantly contribute (or fully mediate) a test compound’s (developmental) neurotoxicity. The best-known representatives of this group are organothiophosphates like parathion [11]. Besides this, the knowledge based on group (iii) is still very limited and needs expansion, possibly based on new test data. For the overall risk assessment, one needs to keep in mind that the derived potency ratios measured here may only provide a theoretical upper bound of what is possible. In many cases, in vivo phase II metabolism or other pathways are likely to reduce the availability of toxic metabolites.

## Data Availability

Data can be accessed via the biostudies accession number: S-BSST2142.

## Supplementary Materials

The following supporting information can be downloaded at: https://www.mdpi.com/article/10.3390/ijms26178338/s1. References [43,90,91] are cited in the supplementary materials.

## Abbreviations

The following abbreviations are used in this manuscript:

BMC	Benchmark Concentration
BMR	Benchmark Response
cMINC	Neural Crest Cell Migration Assay
CYP	Cytochrome P450 (enzyme family)
DNT	Developmental Neurotoxicity
DNT-IVB	Developmental Neurotoxicity In Vitro Battery
DoM	Day of Migration
EC	Effect Concentration
FMO	Flavin-containing Monooxygenase
G6P	Glucose-6-phosphate
HLM	Human Liver Microsomes
HPP+	Haloperidol Pyridinium Ion
KNDP	Key Neurodevelopmental Processes
LUHMES	Foetal Human Mesencephalic Cell Line
MDMA	Methylenedioxymethamphetamine
MitoMet	Mitochondrial Toxicity Assay Variant of NeuriTox
MPP+	1-methyl-4-phenylpyridinium
MPTP	1-methyl-4-phenyl-tetrahydropyridine
NAM	New Approach Methodology
NCC	Neural Crest Cell
NGRA	Next-Generation Risk Assessment
S9	Post-Mitochondrial Supernatant Fraction
