# Supplementary material for: Testing Strategies for Metabolite-Mediated Neurotoxicity
Source: Int J Mol Sci. 2025 Aug 28;26(17):8338. doi: 10.3390/ijms26178338 (PMC12428754; doi:10.3390/ijms26178338)
Supplement: Supplementary file 1 [file ijms-26-08338-s001.zip › Suess2025_Supplements_V2.pdf]

International Journal of
Molecular Sciences

Supplementary Material

Testing strategies for metabolite-mediated neurotoxicity

Julian Suess[1], Moritz Reinmoeller[1], Viktoria Magel[1], Baiba Gukalov[2], Edgars Liepins[2], Iain Gardner[3], Nadine Dreser[1,4], Anna-Katharina Holzer[1], Marcel Leist[1,4,*]

Table of contents

Element	Page	Title
Fig. S1	1	Comparison of data from the MitoMet test and the PeriTox test.
Fig. S2	2	Neurotoxicity hazard identification of selected parent/metabolite pairs using cMINC.
Fig. S3	3	Quantification of specific metabolite formation from selected parent/metabolite pairs in a human liver microsomes (HLM) assay.
Table S1	4	Prediction of free fraction in conditions with or without S9 of selected parent/metabolite pairs.
Table S2	4	MRM transitions and MS parameters for analytes.
Table S3	5	Overview of issues encountered, when establishing an S9-based workflow for DNT assays.
Supp. File S1	NA	Additional compound identifiers and physchem properties.

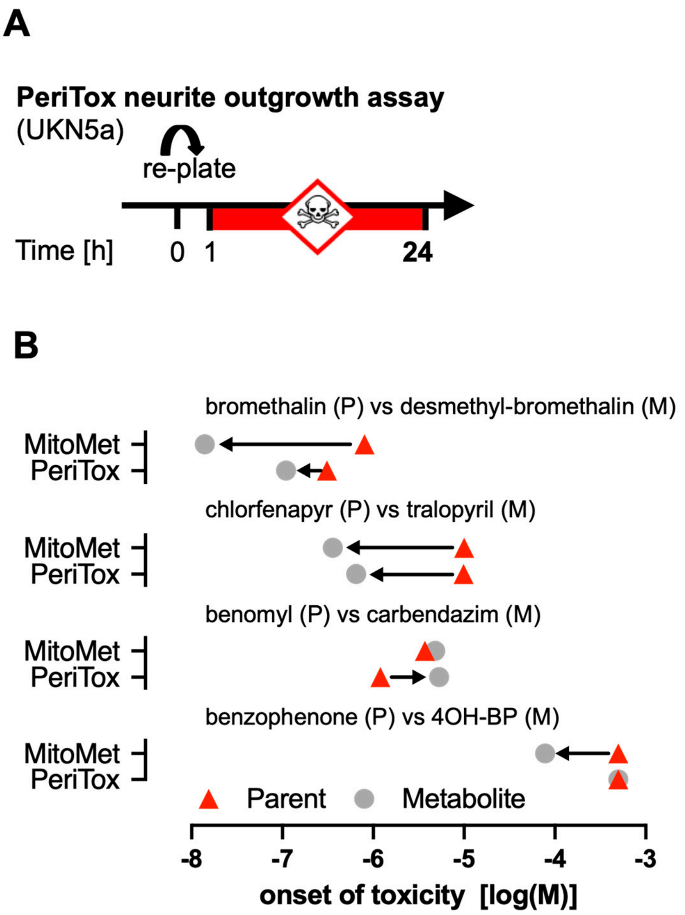

Supplementary Figure S1: *Comparison of data from the MitoMet test and the PeriTox test.* A) *Exposure scheme for the PeriTox test (UKN5a): For testing, cryopreserved hiPSC-derived immature human dorsal root ganglia neuron-like cells were thawed and plated into 96-well plates. Cells were treated with toxicants at one h after plating. Neurite outgrowth and viability were determined at 24 h later, using automated high content imaging. B) Benchmark concentrations (BMC25) were calculated as a measure for "onset of toxicity" for the MitoMet (UKN4b; as described in Figure 1) and UKN5a assay. If no BMC could be determined, the highest tested concentration was used as surrogate endpoint. The arrows indicate the sensitivity shifts of parent vs metabolite in the respective assay.*

A

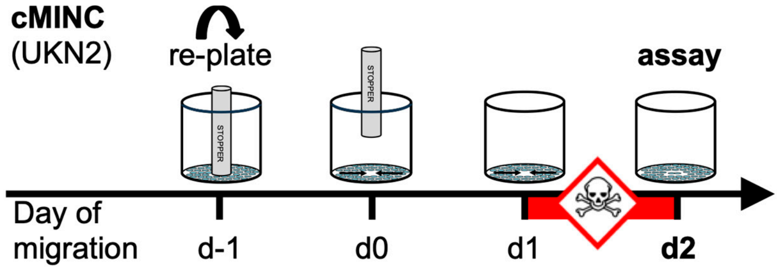

B

compound	BMC25 (migration) [µM]	Ratio (P/M)
(P) Benzophenone	>500	3
(M) 4-Hydroxy-benzophenone	178	
(P) Bromethalin	0.43	44
(M) Desmethyl-bromethalin	0.01	
(P) Chlorfenapyr	4.7	28
(M) Tralopyril	0.17	

Supplementary Figure S2*: Neurotoxicity hazard identification of selected parent/metabolite pairs using cMINC. A) Experimental set up as described in Fig. 6. B) Table of derived benchmark concentrations (BMC25) and parent (P)/metabolite (M) potency ratios.*

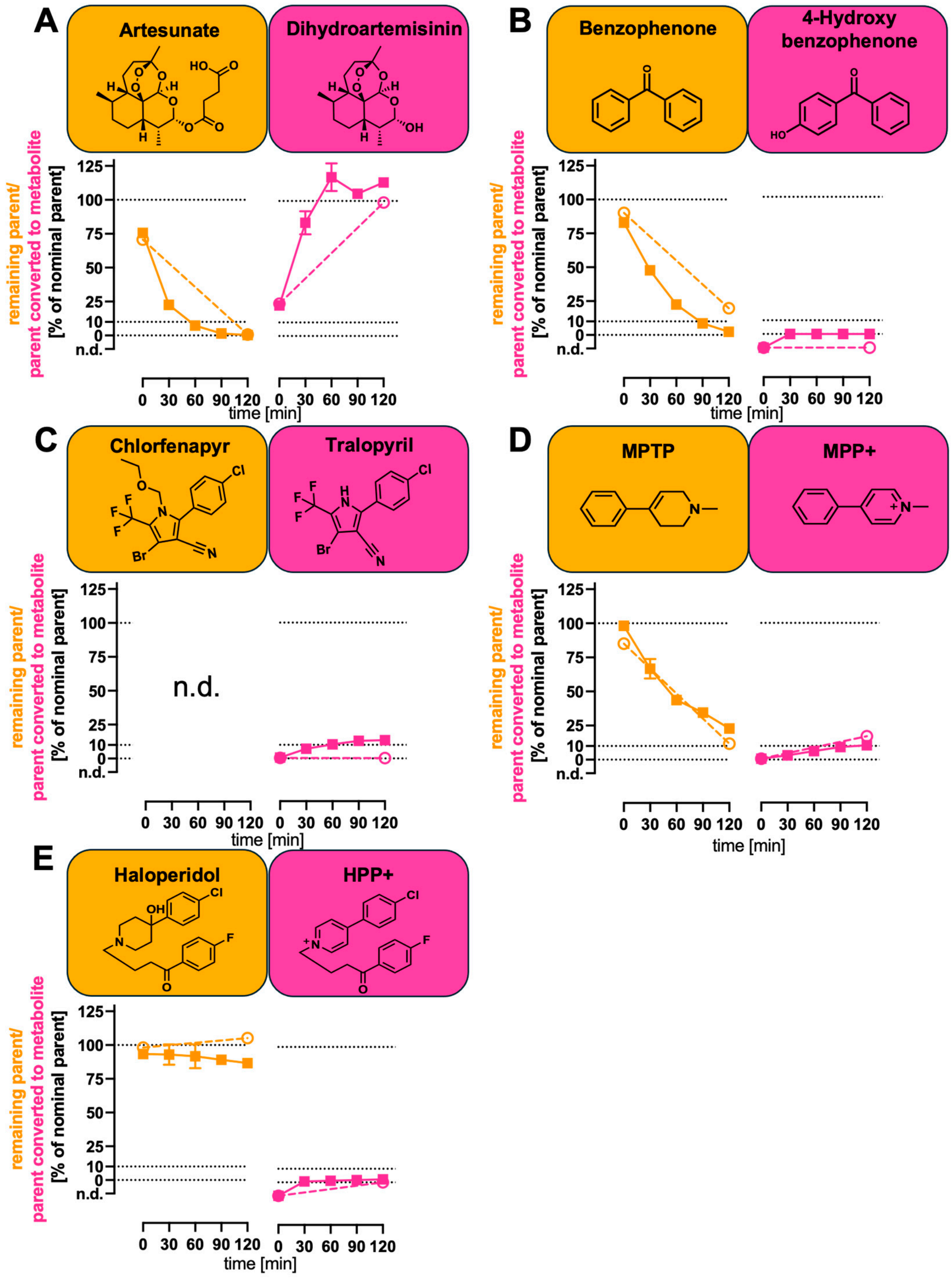

Supplementary Figure S3: *Quantification of specific metabolite formation from selected parent/metabolite pairs in a human liver microsomes (HLM) assay. Parent compounds (PAR) were incubated at a concentration of 5 µM (50 µM for chlorfenapyr) with HLM in the absence (dashed line, open symbols) or presence of NADPH (solid lines, closed symbols). Samples were taken every 30 min for 2 h (+NADPH) or at 2 h only (-NADPH). The remaining fraction of the PAR was quantified for each sample. Additionally, one key metabolite (MET) was measured. Its amount is expressed as fraction of PAR converted to MET, assuming an equimolar PAR to MET reaction, 100 % indicates a complete conversion. The middle-dashed line indicates a metabolite formation of at least 10% of PAR. Data are means of two measurements n.d. = not detected. (**A**)Artesunate degradation and dihydroartemisinin formation. (**B**) Benzophenone degradation and 4-hydroxybenzophenone formation. (**C**) Chlorfenapyr degradation and tralopyril formation. (**D**) MPTP degradation and MPP+ formation. (**E**) Haloperidol degradation and HPP+ formation.* **Abbreviations**: HPP+, haloperidol pyridinium ion; MPP+, 1-methyl-4-phenylpyridinium; MPTP, 1-methyl-4-phenyl-tetrahydropyridine

Supplementary Table S1: *Prediction of free fraction in conditions with or without S9 of selected parent/metabolite pairs*

**Parent**/CAS	**Free fraction [%]**		**Metabolite of concern** CAS	**Free fraction [%]**	
	-S9	+S9		-S9	+S9
**Artesunate** CAS: 88495-63-0	98.2	97.7	**Dihydroartemisinin** CAS: 71939-50-9	99.7	97.2
**Benomyl** CAS: 17804-35-2	99.6	96.9	**Carbendazim** CAS: 10605-21-7	100.0	98.8
**Benzophenone** CAS: 119-61-9	99.4	94.9	**Hydroxybenzophenone** CAS: 1137-42-4	99.2	96.7
**Bromethalin** CAS:63333-35-7	83.2	20.8	**Desmethyl-bromethalin** CAS: 57729-86-9	62.6	5.4
**Chlorfenapyr** CAS: 122453-73-0	96.4	68.2	**Tralopyril** CAS: 122454-29-9	98.4	84.9
**Febantel** CAS: 58306-30-2	99.1	91.6	**Fenbendazole** CAS: 43210-67-9	100.0	80.7
**Haloperidol** CAS: 52-86-8	99.8	96.9	**HPP+** CAS: 125785-69-5	99.9	99.6
**MDMA** CAS: 64057-70-1	100.0	99.5	**α-Methyldopamine** CAS: 555-64-6	100.0	99.9
**MPTP** CAS: 28289-54-5	99.9	98.4	**MPP+** CAS: 48134-75-4	100.0	100.0

Supplementary Table S2 *MRM transitions and MS parameters for analytes.*

Compound	ESI mode	MRM transition	Cone, V	Coll, eV
Artesunate	positive	407.10 > 261.20	10	14
Dihydroartemisinin	positive	307.20 > 261.20	30	8
Benzophenone	positive	183.00 > 104.90	20	15
4-Hydroxybenzophe-none	positive	199.00 > 105.00<br>199.00 > 121.00	20	16
MPTP	positive	174.10 > 43.90	20	10
MPP+	positive	170.00 > 128.00	20	25
Haloperidol	positive	376.10 > 123.00	20	40
		376.10 > 165.10	20	25
HPP+	positive	354.10 > 123.00	10	35
Tralopyril	negative	346.80 > 78.90	10	30
Reserpine	positive	609.20 > 195.20	50	35

Supplementary Table S3: *Overview of issues encountered, when establishing an S9-based workflow for DNT assays***.** *For each issue, the table provides an exemplified solution used in the current context and lists alternative strategies that may also be considered.*

**Issues addressed**	**Solution exemplified here**	**Other potential solutions**
S9 toxicity to cells	Preactivation workflow with subsequent dilution. Plus precleaning: removal of freezing artefacts by centrifugation.	Immobilization of S9 by encapsulation [1] or entrapment [2]
Cofactor toxicity (NADPH) [1]	G6PDH based NADPH re-generation system.	Isocitrate dehydrogenase NADPH re-generation system.
Interference with test method endpoint (e.g. calcein) [1]	Recombinant expression of fluorophore instead of calcein use (MitoMet); complete media exchange before staining (cMINC).	Separation of metabolising system from test system; change of endpoint (not affected by S9)
Binding of test compound/ metabolites to protein	Comparison of S9 +/- cofactors	Inactivated S9 or alternative protein source as the control condition; extraction of metabolite/parent mixture by organic solvent before transfer.
Loss of toxicity by dilution	Starting at higher concentration	Compound enrichment procedures; microfluidics
Optimal incubation time	Pilot studies with positive control(s)	Measurement of metabolite formation.
Identification of positive controls	Use of known parent/metabolite pairs	Exploration of prodrugs; broader screening
"Non-Cyp" metabolism	Exemplified by MPTP (metabolised by monoamine oxidase)	Comparison of S9 metabolism vs adequate control condition; use of specific inhibitors; use of subfractions or isolated enzymes.
Formation of reactive (short lived) metabolites	Not considered important, as they play minor roles for neurotoxicity.	Specific trapping assays; continuous flow systems; use of fresh primary human hepatocytes
Phase II metabolism	Not addressed; as mostly associated with detoxification	Addition of respective cofactors.
S9 origin	Phenobarbital/β-naphthoflavone-induced male Sprague Dawley rat hepatic S9	Biotechnological metabolisation system like ewoS9R [3];<br>human S9 (multi donor)

Abbreviations*: G6PDH: glucose-6-phosphate dehydrogenase; Non-Cyp metabolism: xenobiotic metabolism by enzymes not belonging to the cytochrome P450 class; MPTP, 1-methyl-4-phenyl-tetrahydro-pyridine.* ***References*** *[1] Deisenroth, C.; DeGroot, D.E.; Zurlinden, T.; Eicher, A.; McCord, J.; Lee, M.Y.; Carmichael, P.; Thomas, R.S. The Alginate Immobilization of Metabolic Enzymes Platform Retrofits an Estrogen Receptor Transactivation Assay with Metabolic Competence. Toxicological Sciences 2020, 178, 281–301, doi:10.1093/toxsci/kfaa147; [2] Riley, R.; Roberts, P.; Coleman, M.; Kitteringham, N.; Park, B. Bioactivation of Dapsone to a Cytotoxic Metabolite: In Vitro Use of a Novel Two Compartment System Which Contains Human Tissues. Br J Clin Pharmacol 1990; [3] Brendt, J.; Lackmann, C.; Heger, S.; Velki, M.; Crawford, S.E.; Xiao, H.; Thalmann, B.; Schiwy, A.; Hollert, H. Using a High-Throughput Method in the Micronucleus Assay to Compare Animal-Free with Rat-Derived S9. Science of the Total Environment 2021.*
